# A proposed novel adaptive DC technique for non-stationary data removal

**DOI:** 10.1016/j.heliyon.2023.e13903

**Published:** 2023-02-21

**Authors:** Hmeda Musbah, Hamed H. Aly, Timothy A. Little

**Affiliations:** Department of Electrical and Computer Engineering, Dalhousie University, Halifax, Canada

## Abstract

The stationarity of a time series is an important assumption in the Box-Jenkins methodology. Removing the non-stationary feature from the time series can be done using a differencing technique or a logarithmic transformation approach, but it is not guaranteed from the first step. This paper proposes a new adaptive DC technique, a novel technique for removing a non-stationary time series from the first step. The technique involves transferring non-stationary data into another domain that deals with it as a stationary time series, as it is much easier to be forecasted in that domain. The adaptive DC technique has been applied to different time series, including gasoline and diesel fuel prices, temperature, demand side, inflation rate and number of internet users time series. The performance of the proposed technique is evaluated using different statistical tests, including Augmented Dickey-Fuller (ADF), Kwiatkowski-Phillips-Schmidt-Shin (KPSS), and Phillips Perron (PP). Additionally, the technique is validated by comparing it with a differencing technique, and the results show that the proposed technique slightly outperforms the differencing method. The importance of the proposed technique is its capability to get the stationarity data from the first step, whereas the differencing technique sometimes needs more than one step.

## 1 Introduction

**Fig. 1:**
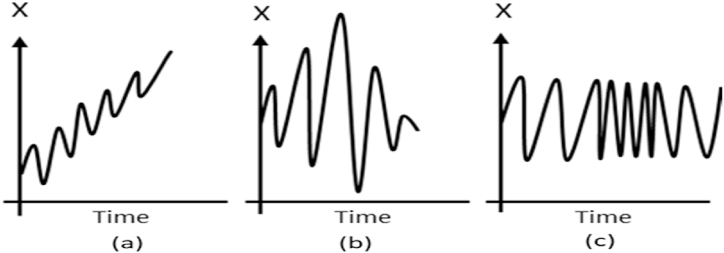
Various cases of non-stationary data.

Various studies have used different models to forecast electricity prices, demand-side, and stock market prices. One of the most used techniques is the Autoregressive Integrated Moving Average (ARIMA) due to its simplicity and accuracy [1]. However, the ARIMA model can be used for estimation if the time series of the data is stationary. The term “stationary data” [2] indicates that data mean, covariance, and autocorrelation are constant for a time period of the time series. Figures (1a), (1b), and (1c) show different cases of non-stationarity, namely the mean, covariance, and variance changing, respectively, in the time series over time. For prediction purposes, stationary data are much easier to predicate than non-stationary data. Trend and seasonality are two main components that can convert stationary to non-stationary data and vice versa. These two components play an important role in varying the mean and the variance [3].

**Fig. 2:**
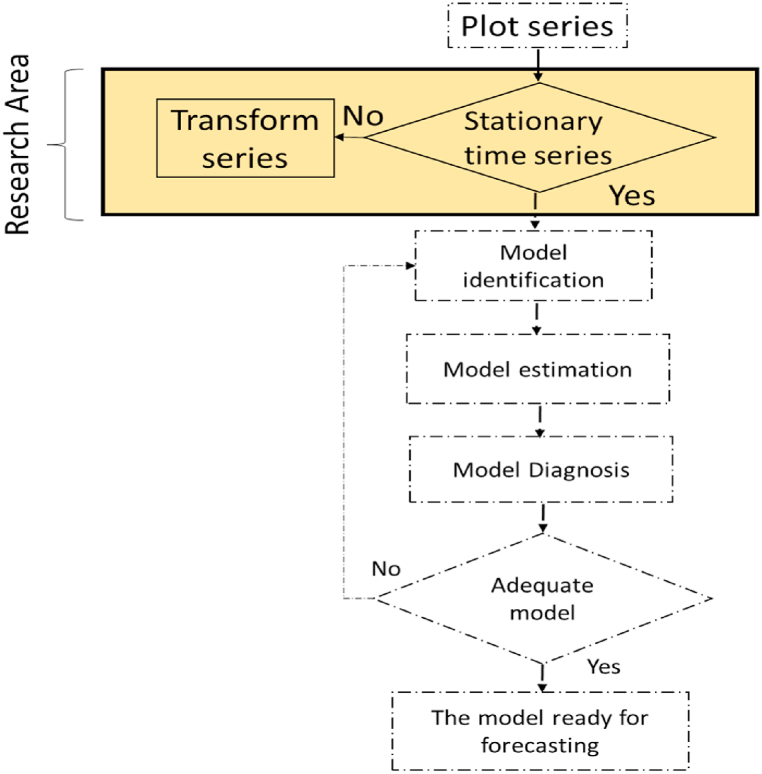
Flowchart of Box-Jenkins steps.

**Fig. 3:**
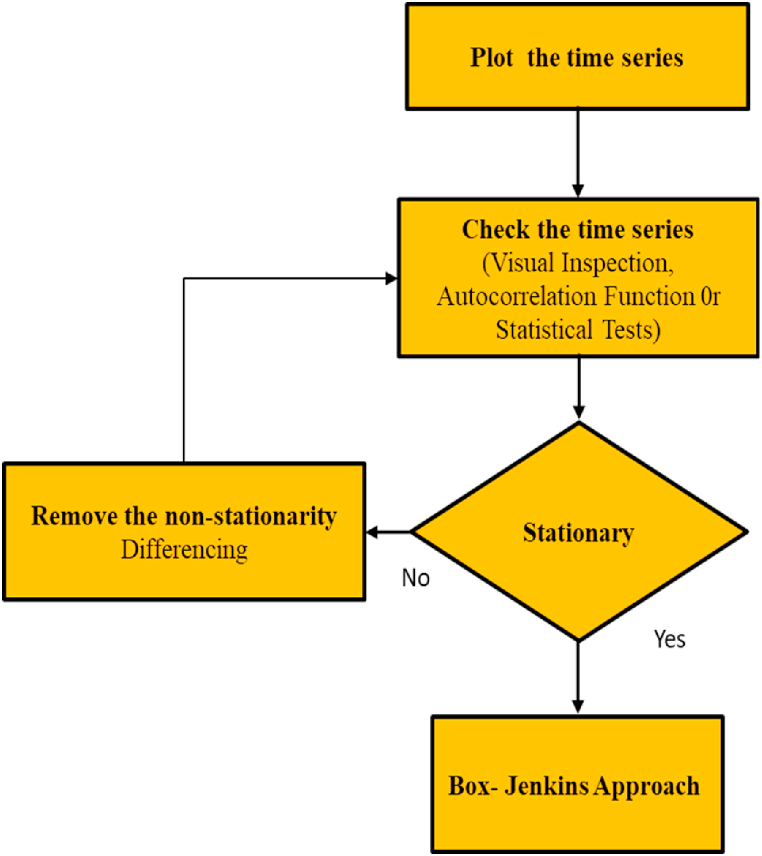
Flowchart showing the first stage in the Box-Jenkins approach.

More specifically, the trend controls the mean, and the seasonality controls the variance. Numerous studies have been conducted to understand whether the data has a trend or seasonal component. The Box-Jenkins approach, as shown in Figure (2), assumes in the first step that the time series should be stationary [2,4]. Checking for stationarity is presented separately in Figure (3). There are two main processes in the stationarity step: detection and transformation. The detection process includes aspects such as visual inspection, autocorrelation function (ACF), statistical tests [5], and (FFT) [6]. This process is concerned with checking whether the time series is stationary.

In a case where the time series is stationary, we proceed to the Box-Jenkins approach. Otherwise, the time series is transformed into stationary, which is called the transformation process. Transformation can be done using a differencing technique or a logarithmic transformation approach, but those approaches are sometimes converting the nonstationary data into stationary one after more than one step, and this will increase the error, particularly if the converted data will be used for prediction after the conversion.

In this work, a novel technique known as an Adaptive DC Technique has been proposed to remove the stationarity from the different time series of gasoline and diesel fuel prices, temperature, demand side, and inflation rate. The advantage of this technique is its capability to remove the non-stationarity from the first step for any kind of data compared to other techniques. The results of the adaptive DC technique are compared to the differencing technique using statistical tests such as the Augmented Dickey-Fuller (ADF), Kwiatkowski-Phillips-Schmidt-Shin (KPSS), and Phillips Perron (PP), and the proposed technique proved its effectiveness.

The novelty of this work is to propose an Adaptive DC technique that will improve the forecasting of nonlinear datasets of most renewables, resulting in the enhancement of reliability. The technique has been implemented in Box- Jenkins approach and used instead of the differencing method. The importance of the proposed technique is its capability to eliminate the nonstationary portion from the first step for a complicated data compared to the differencing method that may need more than one step. The work was validated by comparing it with benchmark work done in that area.

## 2 Literature review

A hybrid model consisting of Auto-Regressive Integrated Moving Average (ARIMA) and Artificial Neural Network (ANN) methods are examined over several datasets. As the Augmented Dickey-Fuller (ADF) test shows, Turkey's intraday electricity market price and the exchange rate between the British pound and the U.S. dollar dataset are non-stationary, so a natural logarithmic function is used to transform them into stationary datasets. The results show that the ARIMA performs well when the dataset is stationary, and ANN models work well with non-stationary data, but if the dataset is stationary, the performance is improved [7]. The ARIMA model was used to forecast small-scale agricultural load. A small-scale agricultural load is non-stationary, so the transformation can potentially be done using differencing, deflating, or logging [8]. Furthermore, in Refs. [9,10], the authors claim that log transformation and differencing are suitable approaches to remove the non-stationarity but maybe more than one step is needed, which is time-consuming and increases the error profile. They use a differencing technique to convert the Johns Hopkins epidemiological data from non-stationary to stationary. Four graphical techniques are applied in Ref. [11] to identify the seasonal component, the authors mentioned that seasonal subseries and box plots have limitations in identifying the seasonal. There are other statistical tests have mentioned in Ref. [12], where they can be used to identify seasonality. Various signal processing techniques are used to detect the seasonality in wind speed time series by applying Continuous Wavelet Transform (MCWT) [13]. Researchers in Ref. [14] utilize the Box-Jenkins algorithm for forecasting the monthly average surface temperature, they used visual inspection and the ACF for detecting the seasonality. All these methods confirmed the existence of seasonality. Table 1 presents some studies that have been conducted to check and remove stationarity. Non-stationary data is hard to be predicted using ARIMA. Non-stationarity part should be removed first. Time series forecasting techniques such as Neural networks, Wavelet, and a Kalman filtering estimator are utilized widely for forecasting. Many hybrid techniques are proposed in the literature to improve the accuracy of the forecasting models, especially for nonlinear data [15,16].

**Table 1:** A sampling of studies that apply common tools to identify and remove stationarity.

Reference	Year	Time series dataset	Checking/Removing stationarity	Comments
[17]	2003	Electricity prices	Visual inspection/Logarithmic transformation.	• Hard to detect stationarity in long time series but gives a clear view in short time series. • Logarithmic transformation is preferred.
[18]	2009	Load demand time series	Visual inspection/Differencing method.	• Hard to detect stationarity in long time series but gives a clear view in short time series. • Two and one non-seasonal and seasonal differentiation, respectively.
[19]	2012	Solar radiation	Visual inspection and ADF/Differencing method.	• ADF tends to reject the null hypothesis. • First order difference was performed on solar radiation data.
[20]	2012	Weekly rainfall	Autocorrelation Function (ACF) and visual inspection/Logarithmic transformation and differencing method.	• The seasonality must be significant, otherwise the ACF tool cannot detect it. • The stationarity in mean and variance are done by performing log transformation and differencing of the original data.
[21]	2014	Wind speed	Visual inspection/Differencing method.	• Hard to detect stationarity in long time series but gives a clear view in short time series. • First order difference was performed to achieve stationarity.
[2]	2014	Air temperature	ACF, Kwiatkowski-Phillips-Schmidt-Shin (KPSS) and Dickey-Fuller.	• KPSS tends to reject the null hypothesis. • One non-seasonal and one seasonal differentiation, respectively.
[22]	2015	Wind speed	Mentioned to The Augmented Dickey-Fuller (ADF), but applied the visual inspection/Differencing method.	• ADF tends to reject the null hypothesis. One non-seasonal and one seasonal differentiation, respectively.
[23]	2016	Wind speed	Autocorrelation Function (ACF)/Differencing method.	• Seasonality must be significant, or the ACF tool cannot detect it. • Only one differencing.
[24]	2017	Temperature	ACF and ADF/Differencing method.	• ADF tends to reject the null hypothesis. • One seasonal differentiation.
[25]	2017	Air temperature	Visual inspection, ADF and KPSS.	• ADF and KPSS tend to reject the null hypothesis. • One non-seasonal and one seasonal differentiation, respectively.

## 3 Removing non-stationarity

**Fig. 4:**
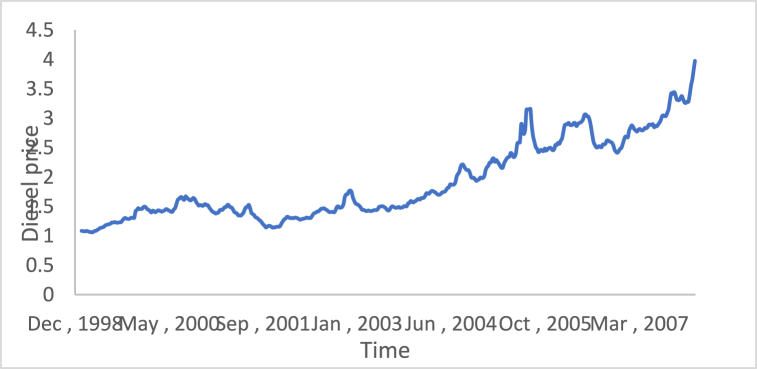
Hourly diesel prices.

**Fig. 5:**
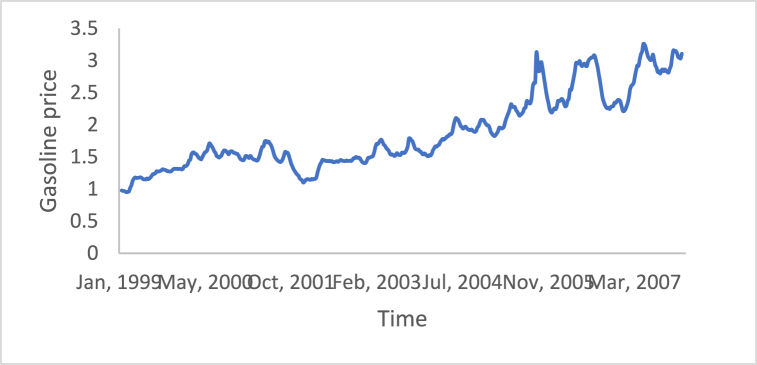
Hourly gasoline prices.

**Fig. 6:**
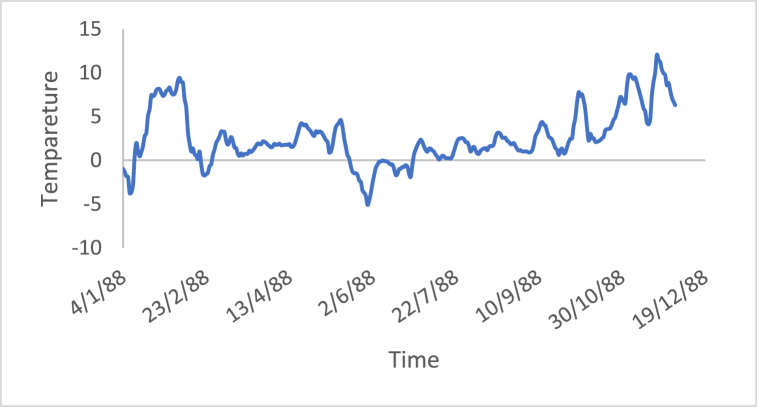
Daily temperature.

**Fig. 7:**
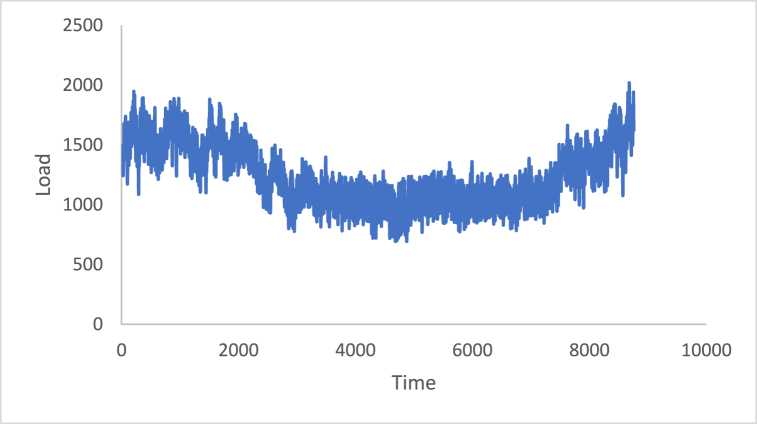
Hourly demand side.

**Fig. 8:**
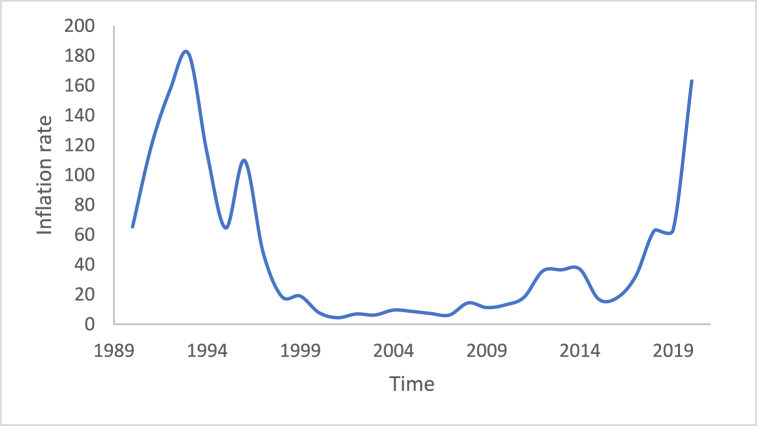
Yearly Inflation rate.

**Fig. 9:**
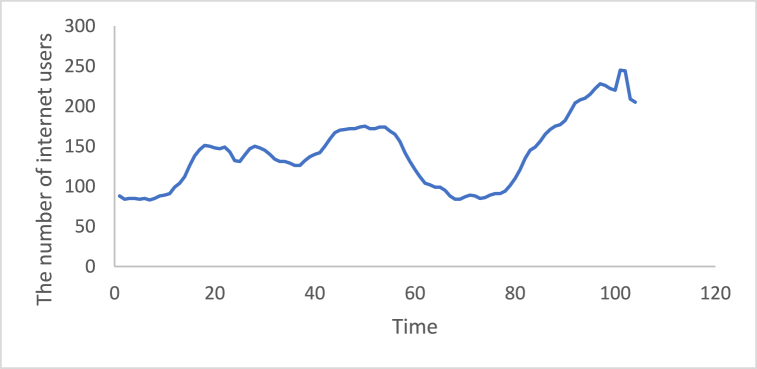
Number of internet users time series.

The stationarity in the time series is a potential condition in Box-Jenkins approach. For this reason, many methods such as differencing and de-trending have been used to achieve this condition. In this study, the differencing technique will be presented, as it is the main tool used in the Box-Jenkins approach in the removing trend. Also, a proposed technique is called Adaptive DC technique is presented in this study. Fig. 4, Fig. 5, Fig. 6, Fig. 7, Fig. 8, Fig. 9 show the time series of hourly diesel prices, hourly gasoline prices, daily temperature, hourly demand side, yearly inflation rate and the number of internet users, respectively. Through using the visual inspection, it is clear the series is non-stationary, because the mean in not constant over time.

### 3.1 Differencing technique

This technique is easy to use but it may be applied more than once to remove the nonstationary part. The differencing equation (1) is written as follows:

Difference

(1) $$\left(t\right)=X_{t}-X_{t-1}$$

where:

$X_{t}\text{:}$ The current observation.

$X_{t-1}\text{:}$ Previous observation**.**

**Fig. 10:**
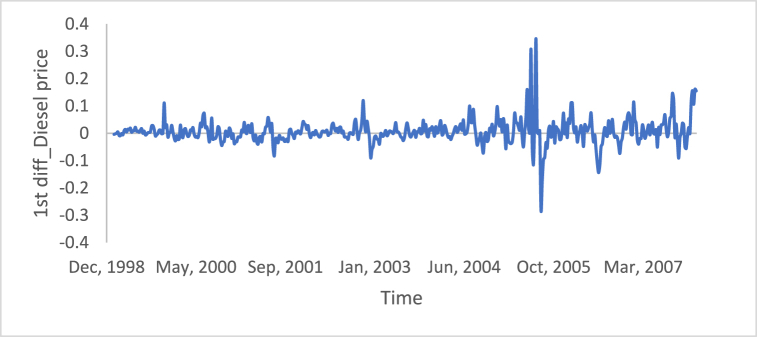
Differencing of the time series of diesel price.

**Fig. 11:**
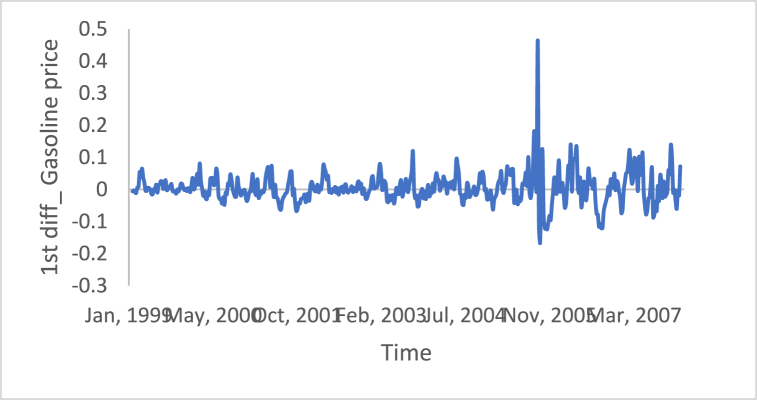
Differencing of the time series of gasoline price.

**Fig. 12:**
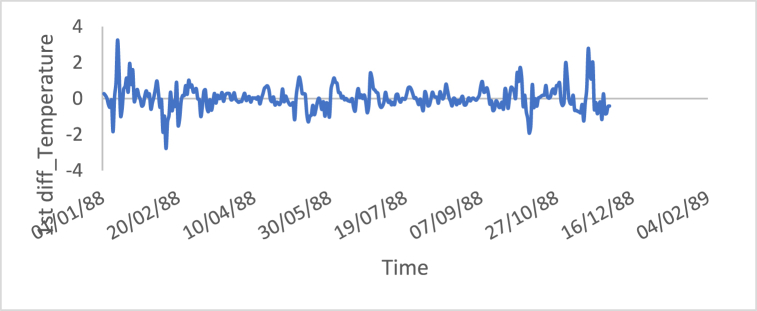
Differencing of the time series of temperature.

**Fig. 13:**
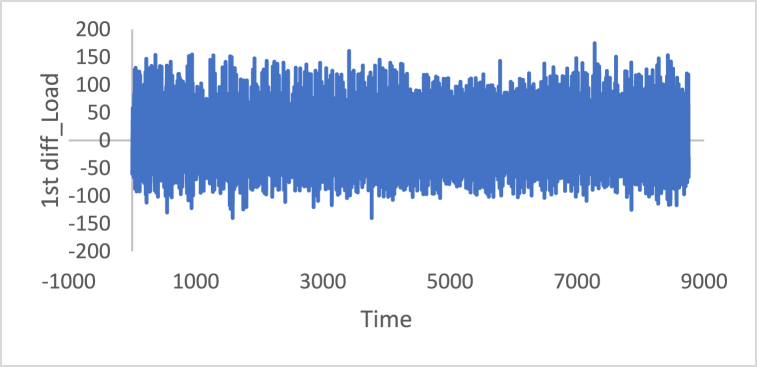
Differencing of the time series of load.

**Fig. 14:**
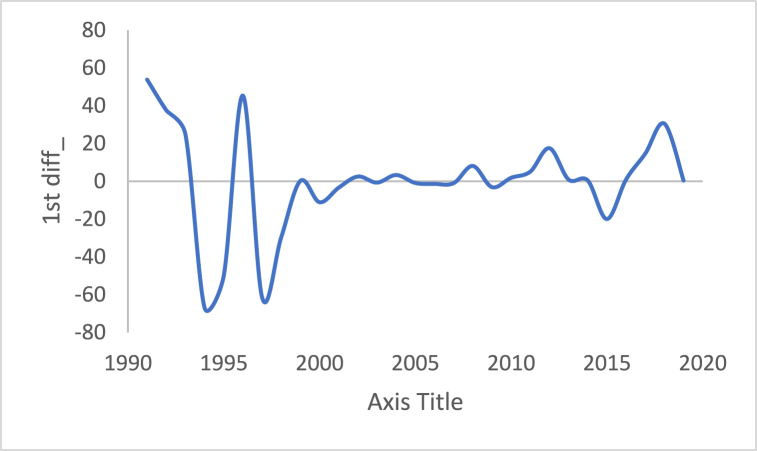
Differencing of the time series of Inflation rate.

**Fig. 15:**
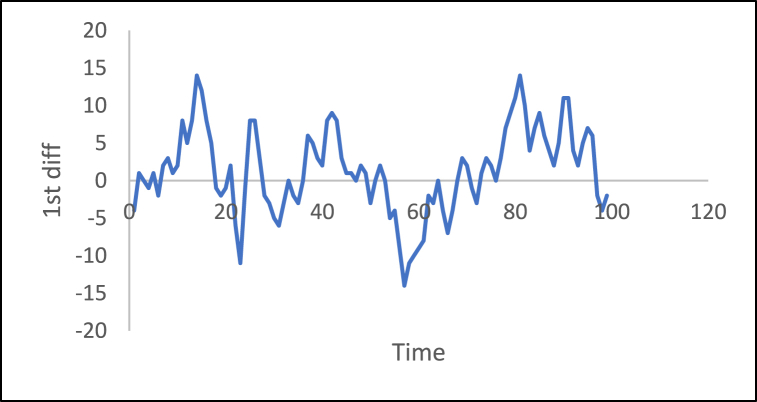
1st Differencing of the time series of the number of internet users.

Fig. 10, Fig. 11, Fig. 12, Fig. 13, Fig. 14, Fig. 15 show the time series of hourly diesel prices, hourly gasoline prices, daily temperature, hourly demand side, yearly inflation rate and the number of internet users, respectively, after applying the first differencing consecutively.

### 3.2 Proposed adaptive DC technique

**Fig. 16:**
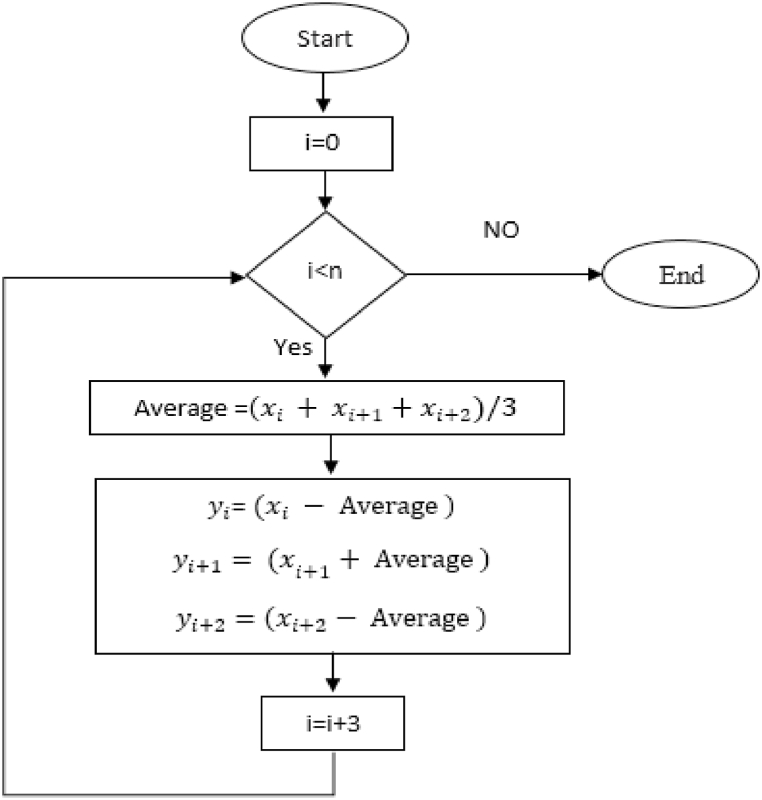
Adaptive DC technique flowchart.

**Fig. 17:**
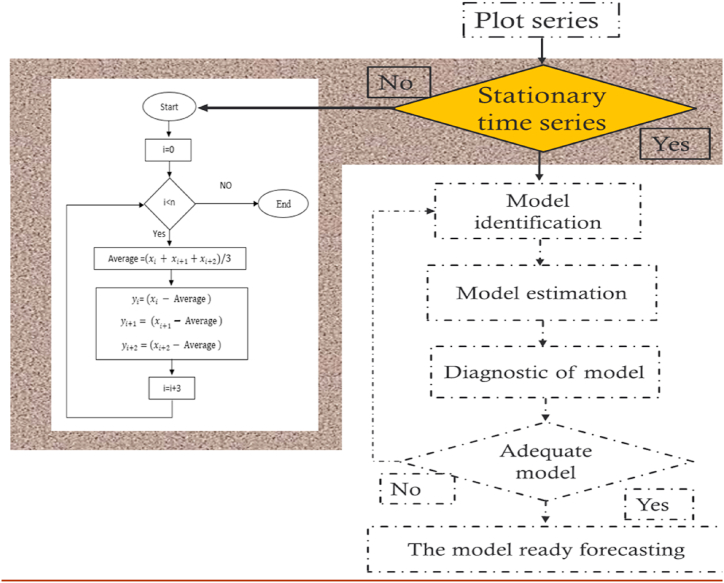
Implementation the Adaptive DC technique in Box-Jenkins approach.

**Fig. 18:**
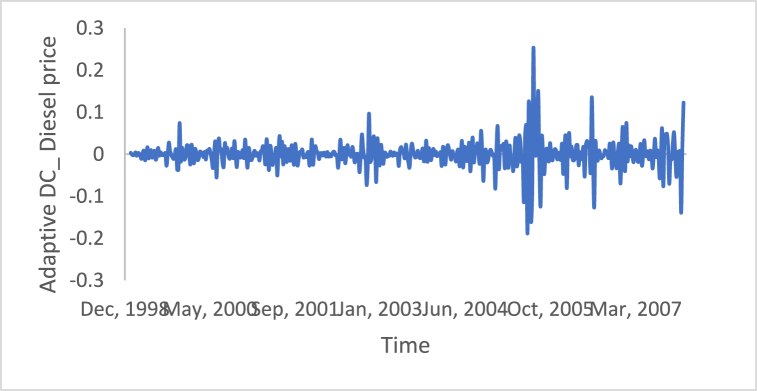
Adaptive DC technique of diesel.

**Fig. 19:**
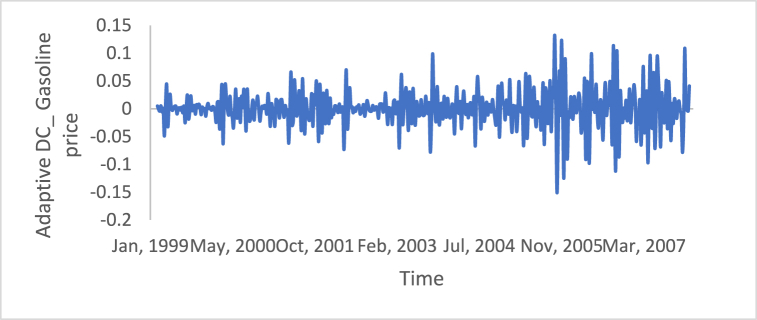
Adaptive DC technique of gasoline.

**Fig. 20:**
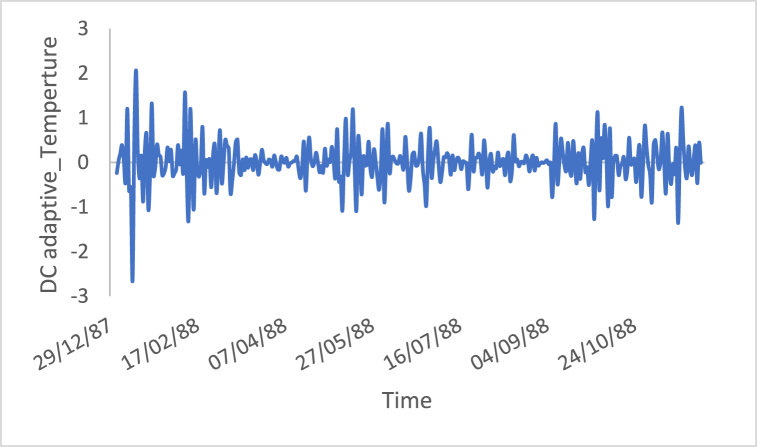
Adaptive DC technique of temperature.

**Fig. 21:**
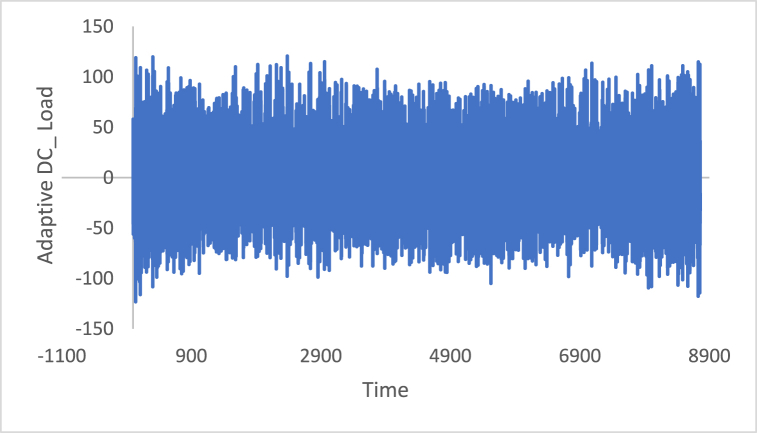
Adaptive DC technique of demand side.

**Fig. 22:**
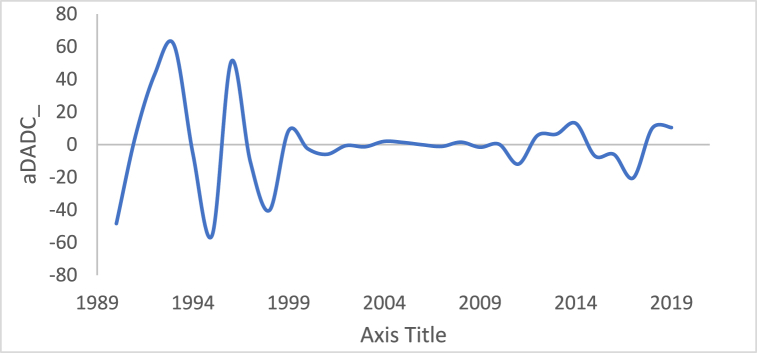
Adaptive DC technique of inflation rate.

**Fig. 23:**
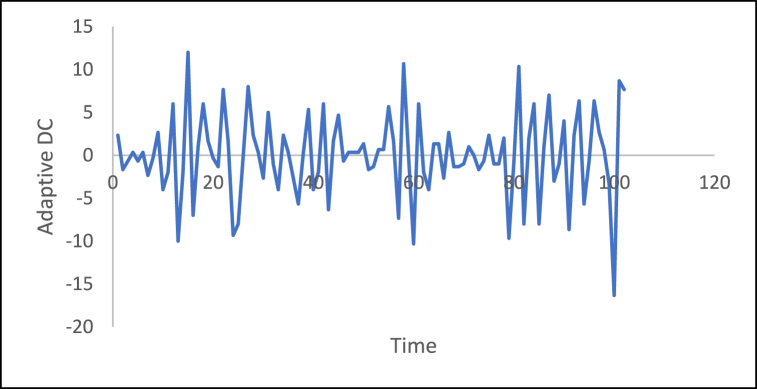
Adaptive DC technique of the number of internet users.

The proposed Adaptive DC technique is a novel approach that can be used to remove trend from a time series. The proposed technique has been inspired by DC offset removal in electrical signals. In the Adaptive DC technique, time series is divided into groups of three points. The mean of the groups is calculated, and every three points are subtracted from the mean. Figure (16) explains the procedure of the Adaptive DC technique flowchart, while figure (17) shows how to implement the Adaptive DC technique in Box-Jenkins approach. The following equation (2) describes it**:**

(2) $$y_{i}=x_{i}-\sum_{j}^{j+2}\left(x_{j}/3\right)$$

where:

$x_{i}\;indicates\;the\;input\;data;\;y_{i}\;denotes\;the$ proposed converted data; i=1;n,j=1,4,7…..n−2; n is the data size; and j changes for each 3i. After performing thousands of runs of the proposed strategy, we found that the best size of j is a pattern of 1,4,7, and so on. The proposed method could work well with different cases of j, but the best performance is found to be when j is defined based on the above pattern. The Adaptive DC technique has been applied to the time series of hourly diesel prices, hourly gasoline prices, daily temperature, hourly demand side, yearly inflation rate and the number of internet users, as shown in Fig. 18, Fig. 19, Fig. 20, Fig. 21, Fig. 22, Fig. 23, respectively.

## 4 Results and discussion

Statistical tests, including ADF, KPPSS and PP, are widely used for checking the stationarity of a time series [26]. The p-values of the tests are compared to 0.05 to decide whether to reject or accept the null hypothesis, where the null hypothesis is non-stationary. In ADF and PP, if p ≥ 0.05, the null hypothesis is true. For p < 0.05, the value is significant enough to reject the null. A key difference between the ADF test and the KPSS test is that the null hypothesis of the KPSS test in the series is stationary.

Table 2 shows that the five-time series are non-stationary. The ability of the statistical tests to identify the stationarity in the time series depends on the time series length [26]. ADF and PP are appropriate tests when the time series length is 25 observations.

**Table 2:** Results of Statistical tests of time series.

Time series	Length of time series	ADF test	KPPSS test	PP test	Status of time series
Diesel prices	467	0.8839	0.01	0.9069	Nonstationary
Gasoline prices	467	0.14	0.01	0.2051	Nonstationary
Temperature	336	0.176	0.01	0.138	Nonstationary
Load	8760	0.656	0.01	0.1	Nonstationary
Inflation rate	31	0.448	0.1	0.9	Nonstationary

Table 3 shows statistical test results after applying the differencing method. The ADF, KPPSS, and PP tests indicate that diesel prices, gasoline prices, and temperature time series are stationary, while the results of the KPPSS test contradict the results of the ADF and PP tests. Furthermore, the KPPSS test confirms the stationarity of the inflation rate time series, whereas the ADF and PP tests show that the inflation rate time series is non-stationary. In this case, the inflation rate time series is considered a non-stationary time series, as its length is 30 observations, and the second differencing is mandatory.

**Table 3:** Results of Statistical tests after applying differencing technique.

Time series	Length of time series	ADF test	KPPSS test	PP test	Status of time series
Diesel prices	467	0.01	0.1	0.01	Stationary
Gasoline prices	467	0.01	0.1	0.01	Stationary
Temperature	336	0.01	0.1	0.01	Stationary
Load	8760	0.01	0.1	0.01	Stationary
Inflation rate	31	0.31	0.1	0.011	Stationary
Number of internet users	105	0.4	0.022	0.8	Non-Stationary

**Fig. 24:**
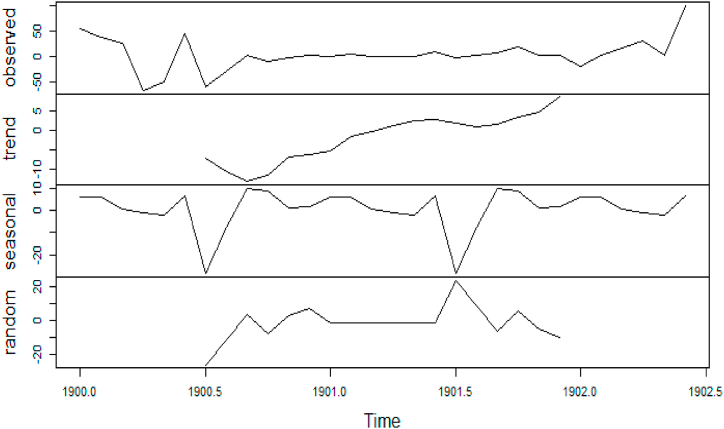
Trend and seasonality decomposition of time series.

**Fig. 25:**
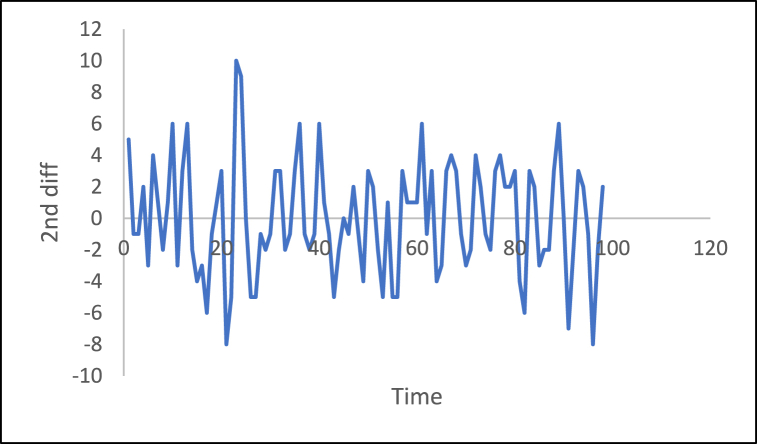
2nd Differencing of the time series of the number of internet users.

In additional investigations, the inflation rate time series is decomposed to check whether the trend component exists, as shown in Figure (24). It is clear from this figure that the trend component is present in the time series, which means the inflation rate time series. This result corresponds to the results of the ADF and PP tests. Figure (15) shows the number of internet users time series after applying the first differencing method. It is hard to evaluate whether the series is stationary through visual inspection. Therefore, the series is split into three contiguous sequences, then we can calculate the mean of each group of numbers and compare the values. The results clearly imply that the mean of the three contiguous sequences is considerably different from each other describing the series is non-stationary. Also, the statistical tests, the ADF, KPPSS, and PP tests, indicate that the series is non-stationary as shown in Table 2. Therefore, the second differencing is mandatory. Figure (25) shows the number of internet users time series after applying the second differencing. It is clear from visual inspection that the number of internet users time series is converted and became stationary.

The ability of differencing method in removing the stationarity from a time series has compared to the proposed method. Through visual inspection, it is found that the six-time series is stationary because their mean and the variance are very constant over time, Fig. 18, Fig. 19, Fig. 20, Fig. 21, Fig. 22, Fig. 23. The ADF, KPPSS, and PP tests confirmed this results, Table 4.

**Table 4:** Results of statistical tests after applying the Adaptive DC technique.

Time series	Length of time series	ADF test	KPPSS test	PP test	Status of time series
Diesel prices	467	0.01	0.1	0.01	Stationary
Gasoline prices	467	0.01	0.1	0.01	Stationary
Temperature	336	0.01	0.1	0.01	Stationary
Load	8760	0.01	0.1	0.01	Stationary
Inflation rate	31	0.01	0.1	0.01	Stationary
Number of internet users	105	0.01	0.1	0.01	Stationary

## 5 Conclusion

This work, a novel Adaptive DC technique was proposed to convert a non-stationary time series to a stationary one from the first step. The adaptive DC technique was applied to various time series of different lengths. The differencing technique was also applied to the same series as a way of validation. The results of the Adaptive DC technique and differencing technique were compared using the ADF, KPSS, and PP tests. The comparison did not show any significant difference when applying both techniques to gasoline and diesel fuel price, temperature, and demand side time series. However, when applying them to inflation rate and number of internet users time series, it was found that the Adaptive DC technique can eliminate the non-stationary portion from the first step compared to differencing technique.

In comparison, the differencing technique may need more than one step. This makes the Adaptive DC technique superior to the differencing technique. The proposed technique reduces the number of steps that minimizes the processing time. As the data became more complicated the other techniques require more steps for the conversion compared to the proposed technique and this will increase the total error for the forecasted data.

## Author contribution statement

Hmeda Musbah: Performed the experiments; Analyzed and interpreted the data; Wrote the paper. Hamed H. Aly: Conceived and designed the experiments; Contributed reagents, materials, analysis tools or data. Timothy A. Little: Contributed reagents, materials, analysis tools or data.

## Funding statement

This research did not receive any specific grant from funding agencies in the public, commercial, or not-for-profit sectors.

## Data availability statement

The data that has been used is confidential.

## Declaration of interest's statement

The authors declare no conflict of interest.
